# Split‐dose 1 L polyethylene glycol (PEG) with ascorbate is non‐inferior to split‐dose PEG with sodium picosulfate and magnesium citrate with similar tolerability: a randomized study

**DOI:** 10.1002/jgh3.12626

**Published:** 2021-08-17

**Authors:** Kumanan Nalankilli, David J Gibson, Shahzaib Anwar, Danny Con, Helen Chen, Robyn Secomb, Peter Gibson, Gregor Brown

**Affiliations:** ^1^ Department of Gastroenterology Alfred Health Melbourne Victoria Australia; ^2^ Department of Gastroenterology Eastern Health Melbourne Victoria Australia; ^3^ Department of Medicine, Monash University Central Clinical School Melbourne Victoria Australia

**Keywords:** bowel preparation, colonoscopy, Plenvu, Prepkit‐C, split‐dose

## Abstract

**Background and Aim:**

Post‐marketing studies comparing low‐volume polyethylene glycol (PEG)‐based regimens are limited. This randomized study aimed to compare the efficacy and tolerability of a novel 1‐L low‐volume PEG‐based preparation: 1 L PEG+Asc (PEG3350, sodium ascorbate, sodium sulfate, ascorbic acid, sodium chloride, and potassium chloride) with PEG+SPMC (PEG3350, sodium chloride, potassium chloride and sodium sulfate, sodium picosulfate, magnesium oxide, citric acid, and aspartame), prior to routine colonoscopy at an Australian tertiary referral center.

**Methods:**

Outpatients undergoing colonoscopy were randomized to receive either split‐dose 1 L PEG+Asc or split‐dose PEG+SPMC. Bowel preparation quality using the Boston Bowel Preparation Scale (BPPS), modified Aronchick scores, procedure time, cecal intubation, and adenoma detection rates were recorded. Patient compliance and tolerability were captured using a standardized questionnaire.

**Results:**

A total of 173 patients were randomized, of whom 164 completed the study and were allocated to 1 L PEG+Asc (*n* = 82) or PEG+SPMC (*n* = 82). Non‐inferiority of 1 L PEG+Asc was demonstrated with 89% achieving successful preparation (total BPPS ≥6 and each sub‐score ≥2) compared with 85.4% in the PEG+SPMC group, resulting in an estimated difference of 3.7% (95% CI −6.6% to 13.9%). The median BBPS was non‐inferior in all colonic segments with 1 L PEG+Asc (BBPS 3 [interquartile range 2–3]) *vs* PEG+SPMC (BBPS 2 [interquartile range 2–3]). More 1 L PEG+Asc patients reported moderate to severe nausea (*P* = 0.028), but overall tolerability was similar.

**Conclusions:**

The quality of bowel preparation achieved with 1 L PEG+Asc is non‐inferior to that with PEG+SPMC, with similar tolerability outcomes. Further studies are required in patients at risk of suboptimal bowel preparation.

## Graphical abstract

This randomized Australian single‐center colonoscopist‐blinded study shows that 1 L PEG+Asc is non‐inferior to PEG+SPMC in achieving successful bowel preparation defined as a BBPS of = 6. In this study, 1L PEG+Asc also demonstrated non‐inferiority in several secondary endpoints such as proportion achieving good‐to‐excellent preparation according to the modified Aronchick scale and demonstrated similar patient‐reported tolerability.

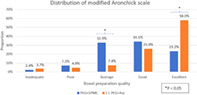

## Introduction

The quality of bowel preparation achieved is critical in determining the quality and completeness of colonoscopy.^1^, ^2^ It is closely associated with other key colonoscopy performance indicators such as the cecal intubation and adenoma detection rate (ADR).^1^, ^3^ Poor bowel cleanliness correlates with prolonged and incomplete procedures, and missed pathology, especially in the right colon.^1^, ^4^ The American Society of Gastrointestinal Endoscopy (ASGE) recommends that a colonoscopy service should target adequate bowel preparation quality in >85% of patients.^5^ More recently, European guidelines have suggested that even higher rates should be targeted, and that a minimum standard of 90% should be maintained.^6^

Although large prospective studies have showed that the quality of bowel preparation has improved over the last two decades,^7^ discomfort associated with consumption of large volumes of bowel preparation regimens is known to adversely impact patients' willingness to undergo a colonoscopy.^8^, ^9^ Therefore, measures to improve the efficacy, safety, and tolerability of bowel preparation regimens continue to be of relevance to colonoscopy services. Recent innovations in this regard are the development of low‐volume polyethylene glycol (PEG) based regimens, split‐dose regimens, and a low‐residue diet such as the “white diet” leading up to colonoscopy.^5^, ^6^, ^10^, ^11^

The recent advent of a split lower‐volume (1‐L in total) PEG and ascorbate‐based preparation (1 L PEG+Asc) has been proposed as a step forward in achieving a balance between efficacy and patient tolerability.^12^, ^13^ However, despite promising results from industry‐sponsored clinical trials,^14^, ^15^, ^16^, ^17^, ^18^ there is a need for post‐marketing real‐world data to further clarify these results.^12^ Furthermore, studies that have directly compared two different low‐volume PEG‐based regimens are limited. The aim of this single‐center randomized study was to compare the efficacy, safety, and tolerability of split‐dose 1 L PEG+Asc ([PLENVU] PEG3350, sodium ascorbate, sodium sulfate, ascorbic acid, sodium chloride, and potassium chloride) with a split‐dose PEG and sodium picosulfate/magnesium citrate formulation PEG+SPMC ([Prepkit‐C] PEG3350, sodium chloride, potassium chloride and sodium sulfate, sodium picosulfate, magnesium oxide, citric acid, and aspartame), in a real‐world population of colonoscopy outpatients.

## Methods

This was a prospective, randomized, colonoscopist‐blinded, non‐inferiority study conducted at a large Australian tertiary hospital. The study protocol was approved by the local ethics committee at our center (Local ethics approval number: 492/17) and conformed to the Declaration of Helsinki. The trial was registered prospectively with the Australia and New Zealand clinical trials registry (Trial ID: ACTRN12618000538246).

### Inclusion criteria

Consenting adult patients (aged ≥18 years) undergoing outpatient colonoscopy for clinically accepted indications were included. These encompassed, but were not restricted to iron deficiency anemia, surveillance of bowel polyps, colorectal cancer screening following a positive fecal occult blood test (FOBT) or a positive family history, assessment of symptoms such as abdominal pain, diarrhea, and constipation, assessment or investigation of inflammatory bowel disease.

### Exclusion criteria

Patients with significant renal impairment (eGFR <30), significant heart failure (New York Heart Association Class III or IV), insulin‐dependent diabetes mellitus, phenylketonuria (due to the presence of aspartame in PEG+SPMC), glucose‐6‐phosphate dehydrogenase deficiency (due to the presence of ascorbic acid in PEG+Asc), a known hypersensitivity to a constituent of PEG+SPMC or PEG+Asc, or previous colonic resection were excluded. Furthermore, patients who have previously had a failed colonoscopy due to poor preparation were excluded.

### Enrolment

All recruitment was undertaken by one of three endoscopy fellows (KN, DG and SA), over a 19‐month period between July 2018 and Jan 2020. Consecutive colonoscopy outpatients booked on endoscopy lists at our center were contacted systematically over the phone, approximately 3–4 weeks ahead of their allocated procedure date. If the patient did not answer the phone, a voice message was left to ring back. If there was no contact back from the patient, a second attempt was made either at the end of the working day or the following day. In addition, limited face‐to‐face recruitment also occurred through the endoscopy outpatient clinic. Patients who verbally agreed to participate were sent a patient information and consent form (PICF), which they read, signed, and returned via prepaid postage.

### Randomization

Participants proceeded to randomization after a signed PICF was received by the study coordinators (HC and RS), who were not involved in either the recruitment process or the colonoscopy procedure. A computer‐generated simple randomization sequence was prepared and stored in a password‐protected file, which was accessible only to the study coordinators. Consecutive participants who returned their signed PICF were randomly assigned to one of two groups–group one (control group): split‐dose PEG+SPMC and group two: split‐dose 1 L PEG+Asc (Table 1). Following randomization, all enrolled participants were either mailed their allocated bowel preparation product or collected this in person. They were given written bowel preparation instructions and were asked to follow a “white diet”^10^, ^19^ for 2 days prior to their colonoscopy. The white diet consists of a range of low‐residue white or cream‐colored foods, such as milk, white yoghurt, and white bread. Since the volume of preparation was different between the two study arms, patients were able to drink approved clear fluids in addition to the actual volume of bowel preparation fluid.

**Table 1:** Bowel preparation regimens and timing

PEG + SPMC (1 sachet of PEG and 2 sachets of SPMC)	Constituents	1 L PEG + Asc (2 Doses)	Constituents
PEG sachet (reconstituted to 1 L)	52.9 g PEG, 2.6 g sodium chloride, 0.74 g potassium chloride, 5.6 g sodium sulfate, and 6 g ascorbic acid	First dose (reconstituted to 500 mL)	100 g of PEG3350, 9.0 g sodium sulfate, 2 g sodium chloride, 1 g potassium chloride, 0.79 g sucralose
SPMC sachet (reconstituted to 250 mL)	10 mg sodium picosulfate, 3.5 g magnesium oxide, 12 g anhydrous citric acid, and 36 mg aspartame	Second dose (reconstituted to 500 mL)	Sachet A: 40 g of PEG3350, 3.2 g sodium chloride, 1.2 g potassium chloride Sachet B: 48.1 g sodium ascorbate, 7.5 g ascorbic acid, 0.875 g aspartame, 1.74 g citric acid flavorings
Morning procedures (0830–1200)	1 sachet of PEG (at 18:00 h) and 1 sachet of SPMC (at 20:00 h) the previous evening, and the second sachet of SPMC consumed at 4:00 h		First dose at 18:00 h the evening prior and the second dose at 4:00 h
Afternoon procedures (1330–1700)	1 sachet of PEG (at 18:00 h) and 1 sachet of SPMC (at 20:00 h) the previous evening, and the second sachet of SPMC consumed at 7:00 h		First dose at 18:00 h the evening prior and the second dose at 7:00 h

### Blinding

All colonoscopies were either performed by a consultant gastroenterologist, or by direct supervision of a gastroenterology trainee by a consultant gastroenterologist. Each participant was advised not to disclose which preparation regimen they had undertaken to the endoscopist during the consent process. Sealed envelopes containing questionnaires specific to the allocated preparation were prepared by the study coordinators and provided to the participants to complete prior to their procedure on the day. Once completed, they were collected and stored. The endoscopist and personnel involved in recruitment were blinded to this information.

### Outcome measures

The primary outcome measure was the proportion of patients achieving successful bowel preparation. This was defined as an overall Boston Bowel Preparation Scale (BBPS) score ≥6,^20^, ^21^ with a score of 2 or more for each bowel segment. The BPPS assesses the quality of bowel preparation in three colonic segments (right, transverse, and left colon) and is scored out of 3, with a total score out of 9. A score of 0 is unprepared, and a score of 9 is entirely clean. If an endoscopist aborted a procedure because of inadequate preparation, then any non‐visualized proximal segments were assigned a score of 0.

Secondary outcome measures included proportion of patients achieving total BPPS = 9, proportion achieving good‐to‐excellent preparation quality as assessed by the modified Aronchick scale,^22^ patient acceptance, compliance, and tolerability. Additional exploratory analyses included the difference in quality of cleansing between morning and afternoon procedures as these have different bowel preparation regimen timings.^23^ Furthermore, any differences in parameters such as the withdrawal time (in non‐polypectomy colonoscopies), the total procedure time,^5^, ^24^ ADR,^4^, ^5^, ^25^ and the cecal intubation rate were evaluated.^1^

### Sample size and statistical analysis

The sample size was calculated assuming an 80% bowel preparation success rate for patients undergoing colonoscopy and a non‐inferiority margin of 15%, based on previous non‐inferiority bowel preparation studies at our center.^10^, ^19^ A total sample size of 300 patients was calculated (150 per arm) with a two‐sided alpha of 5% and power of 80%. An intention‐to‐treat analysis was used to assess the primary outcome. The percentage of success was assessed in each treatment group, and the 95% confidence interval for the difference in success rates was determined using the chi‐square test. Non‐inferiority of the bowel preparation regimen with 1 L PEG+Asc was established if the lower confidence limit for the difference in effect was above negative 15%. In the event non‐inferiority was reached, a test for superiority was made, where superiority was concluded if the two‐sided 95% confidence interval of the difference in success rates excluded 0% (*P* < 0.05). Only the primary endpoint was used to define non‐inferiority or superiority. The Mann–Whitney *U* test was used to compare nonparametric continuous variables in additional exploratory analysis.

In February 2020, recruitment for the study was frozen due to restrictions secondary to the COVID‐19 pandemic in Australia. At that stage, an unplanned interim analysis was performed to determine if further recruitment was required. Statistical analysis was performed in Stata/IC 16 (Texas, USA 2020).

## Results

**Figure 1:**
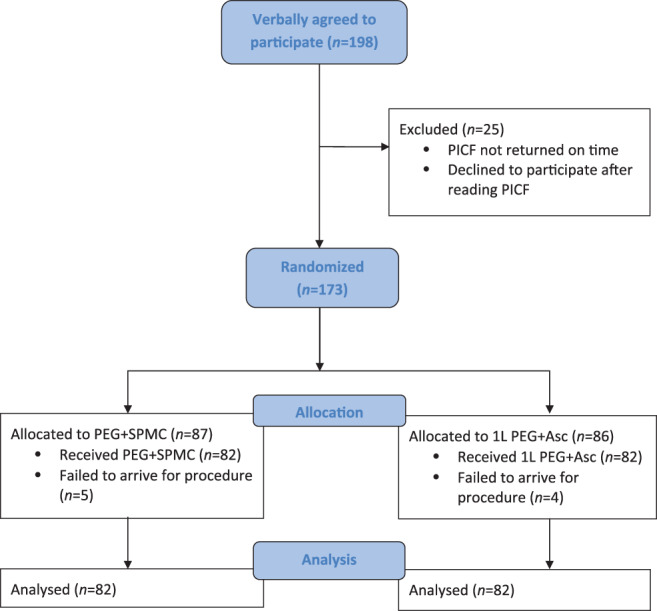
Enrolment flowchart.

A total of 198 participants had been recruited when the study was halted due to the COVID‐19 pandemic. For a variety of reasons, 25 participants failed to return their PICF in time for their procedure. The most common reason for this was delays in the postal service. Of the 173 participants who were randomized, 9 did not arrive for their procedure. This left a total of 164 participants who completed the study (Fig. 1).

The median age of the two groups was similar (PEG+SPMC *vs* 1 L PEG+Asc: 56.7 years *vs* 57.3 years) with a slight male predominance in each group. The indications for colonoscopy were also similar between the two groups, with the most common indications being anemia, per rectal bleeding and/or positive FOBT, or surveillance for previous adenoma or colorectal cancer (Table 2).

**Table 2:** Baseline characteristics of study participants (*n* = 164)

Outcome	PEG + SPMC (*n* = 82)	1 L PEG + Asc (*n* = 82)
Age (years [mean ± SD])	56.7 ± 13.9	57.3 ± 17.5
Male	50 (61)	45 (55)
Indication for colonoscopy (*n* [%])		
Investigation of anemia/per rectal bleed/positive fecal occult blood test	41 (50)	31 (38)
Investigation of cancer (prior polyp/colorectal cancer or family history)	18 (22)	23 (28)
Investigation or monitoring of Inflammatory bowel disease	9 (11)	3 (4)
Investigation of symptoms	7 (9)	14 (17)
Other	7 (9)	10 (12)
Weight, kg, mean ± SD (*n* = 145)	77.7 ± 19.0	80.8 ± 24.8
Height, cm, mean ± SD (*n* = 141)	170.8 ± 11.5	170.5 ± 11.0

The primary endpoint of non‐inferiority was met: 89.0% in the 1 L PEG+Asc group achieved successful preparation (total BPPS ≥6 and each sub‐score ≥2) compared with 85.4% in the PEG+SPMC group, resulting in an estimated difference of 3.7% (95% CI −6.6% to 13.9%). However, criteria to declare superiority were not met (*P* = 0.48).

**Figure 2:**
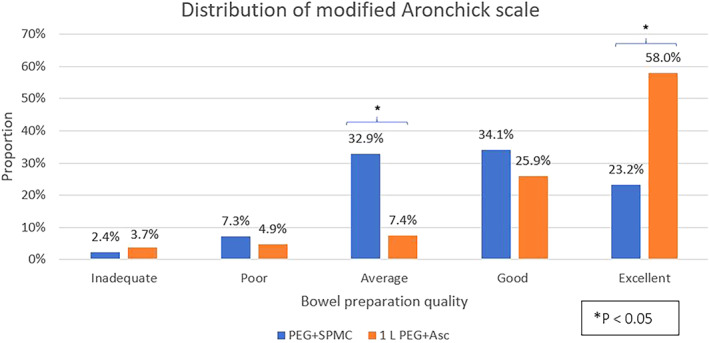
Distribution of modified Aronchick scale

Secondary endpoints were suggestive of superiority; however, given the primary endpoint did not meet criteria for superiority, we could not declare superiority in any of the secondary endpoints. The BBPS sub‐scores were not lower for 1 L PEG+Asc in each of the three colonic subsegments (right, middle, and left colon) compared with PEG+SPMC (median total BBPS 9 *vs* 7; *P* < 0.001) (Table 3). Fifty‐six percent of participants in the 1 L PEG+Asc group had a BBPS of 9 compared with only 27% in the PEG+SPMC group (95% CI 15–44%). The rate of good‐to‐excellent preparation (modified Aronchick scale) was not lower in the 1 L PEG+Asc group compared with the PEG+SPMC group (84% *vs* 57%, 95% CI 13–40%) (Fig. 2).

**Table 3:** Comparison of bowel preparation efficacy between standard PEG+SPMC and 1 L PEG+Asc

Outcome	PEG + SPMC	1 L PEG + Asc	*P*
Boston Bowel Prep Score (BBPS), (median [IQR])			
Left colon	2 (2–3)	3 (2–3)	<0.001
Middle colon	2 (2–3)	3 (3–3)	<0.001
Right colon	2 (2–3)	3 (2–3)	0.001
Total BBPS	7 (6–9)	9 (7–9)	<0.001
Morning procedure	[*n* = 29] 7(6–9)	[*n* = 37] 9(7–9)	
Afternoon procedure	[*n* = 54] 7(6–8) (*P* = 0.56)	[*n* = 46] 9(8–9) (*P* = 0.39)	
Prep success (BBPS ≥6) (*n* [%])	70 (85)	71 (89)	0.48
Total BBPS = 9	22 (27)	45 (56)	<0.001
Procedure time (min)			
Insertion, median (IQR)	6 (5–10)	7 (5–12)	0.37
Withdrawal (non‐polypectomy colonoscopies), median (IQR)	10 (8–12)	10 (8–11)	0.41
Total, median (IQR)	18 (15–23)	20 (15–27)	0.10
Aronchick scale good‐to‐excellent (*n* [%])	47 (57)	68 (84)	<0.001
Cecal intubation (*n* [%])	82 (100)	77 (95)	0.06
Adenoma detection rate (*n* [%])	36(44%)	37(45%)	0.88

There was no difference in the median BBPS between morning and afternoon procedures for both the groups (each *P* > 0.05) (Table 3). Importantly, there were no differences in insertion time, withdrawal time (for non‐polypectomy colonoscopies), or total procedure time between the two groups. ADR (44% [PEG+SPMC] *vs* 45% [1 L PEG+Asc], *P* = 0.88) was high in both groups. Cecal intubation rates were similarly high across the two groups (Table 3).

Tolerability and adverse events relating to each preparation regimen are summarized in Table 4. Vomiting, bloating, and abdominal cramping were similar between the two groups. The only statistically significant difference between the two groups was a higher rate of moderate to severe nausea in association with 1 L PEG+Asc (*n* = 9, 12%) compared with PEG+SPMC (*n* = 2, 2%; *P* = 0.028). However, this did not result in increased rates of vomiting with 1 L PEG+Asc. In particular, 95% of participants in the 1 L PEG+Asc group tolerated 100% of the prep, compared with 89% in the PEG+SPMC group (*P* = 0.16). 86% of participants were willing to repeat the prep in the 1 L PEG+Asc group compared with 90% in the PEG+SPMC group (*P* = 0.47).

**Table 4:** Tolerability and adverse events

Outcome	PEG + SPMC *n* (%)	1 L PEG + Asc *n* (%)	*P*
Accident			
Any	7 (9)	4 (5)	0.54
Vomiting			
Any	4 (5)	9 (11)	0.16
Bloating			
Any	43 (53)	40 (53)	>0.99
Moderate–severe	1 (1)	4 (5)	0.20
Cramping			
Any	33 (41)	37 (47)	0.43
Moderate–severe	4 (5)	3 (4)	>0.99
Nausea			
Any	28 (35)	38 (50)	0.054
Moderate–severe	2 (2)	9 (12)	0.028
Headache			
Any	32 (40)	27 (36)	0.63
Moderate–severe	3 (4)	6 (8)	0.32
Weakness			
Any	31 (38)	33 (44)	0.51
Moderate–severe	4 (5)	5 (7)	0.74
Insomnia			
Any	33 (41)	34 (45)	0.63
Moderate–severe	3 (4)	6 (8)	0.31
Hunger			
Any	48 (59)	38 (49)	0.21
Moderate–severe	5 (6)	8 (10)	0.40
Impact on daily activities			
Any	49 (61)	49 (64)	0.74
Moderate–severe	5 (6)	10 (13)	0.18
Taste, median (IQR)	5 (2–6)	4 (2–7)	0.69
Ease of consumption, median (IQR)	5 (3–8)	5 (3–8)	0.87
Willing to repeat	74 (90)	68 (86)	0.47
Tolerated 100% of prep	71 (89)	77 (95)	0.16
Tolerated ≥75% of prep	78 (98)	81 (100)	0.25

## Discussion

The present study is among the first head‐to‐head post‐marketing randomized studies of 1 L split‐dose PEG+Asc with another well‐established PEG‐based low‐volume split regimen (PEG+SPMC). PEG works as an osmotic laxative that is non‐absorbed in the GI tract. In addition, the PEG+SPMC preparation contains two active ingredients with different mechanisms of action; sodium picosulfate is a stimulant laxative, and magnesium oxide combined with citric acid acts as an additional osmotic laxative.^19^, ^26^ In comparison, the 1 L PEG+Asc preparation contains PEG in both the doses (Table 1). It has a relatively high quantity of ascorbate components,^12^ which results in a higher osmolality load compared with PEG+SPMC. This causes an additional osmotic laxative effect.

In a phase 3 randomized control trial (RCT), split‐dose 1 L PEG+Asc has demonstrated superior efficacy and similar tolerability when compared to 2 L of PEG with ascorbate.^14^ Split‐dose 1 L PEG+Asc has also been shown to be non‐inferior to an oral tri‐sulfate solution.^16^ When administered as a day‐before dosage, 1 L PEG+Asc has demonstrated non‐inferiority to a sodium picosulfate/magnesium citrate (SPMC) regimen,^18^ although in this study, the overall bowel preparation success with 1 L PEG+Asc was reported as only 62%, which suggests that 1 L PEG+Asc may be better used in split‐dosage.

Many studies have previously compared PEG and ascorbate‐based formulations with other low‐volume regimens.^27^, ^28^ However, these were mostly with higher volume 2 L regimens and often showed mixed results. In a RCT of 393 patients that compared split‐dose PEG+Asc as a 2 L formulation (Moviprep) with a SPMC regimen and additional clear fluids, the proportion of patients with very good or good bowel preparation quality was significantly better with 2 L PEG+Asc (98.5% *vs* 57.5%).^29^ Furthermore, the ADR in the right colon was significantly higher (21.0% *vs* 11.9%, *P* = 0.015), as was the detection of flat lesions (21.5% *vs* 13.0%, *P* = 0.025).^29^ In a RCT, 2 L PEG+Asc had similar efficacy but better tolerability when compared with a PEG+SPMC regimen.^30^ However, these results were in contrast with another RCT that compared 2 L split‐dose PEG+Asc (Coolprep) with 2 L of a split‐dose SPMC regimen (Picolight) in 200 outpatients, where the rate of successful bowel preparation (BBPS ≥6) was similar (82% *vs* 80%; *P* = 0.718); however, the SPMC regimen caused fewer gastrointestinal adverse symptoms and tasted better.^31^

In the present study, 1 L PEG+Asc demonstrated non‐inferiority compared with PEG+SPMC using the predefined primary end point for successful bowel preparation, which was a median BBPS of ≥6, with a median score of at least 2 in each segment. The lower‐volume 1 L formulation, in theory, should improve patient acceptability and compliance due to the lower volume of fluid to be consumed. However, no significant differences were observed in tolerability or willingness to repeat the preparation between both groups (Table 4). Despite a lack of superiority observed with the primary endpoint, it did appear that the quality of bowel preparation may have been better using 1 L PEG+Asc. In particular, right‐sided colon cleanliness, which is a major limiting factor for the success of low‐volume preparations, was at least non‐inferior for 1 L PEG+Asc compared with PEG+SPMC. Furthermore, there were no differences in bowel preparation quality within the 1 L PEG+Asc group, between the right and left colon (Table 3). This finding is consistent with other studies that have reported on the efficacy of PEG+Asc based formulations in the right colon.^29^ Furthermore, the higher osmolality load in 1 L PEG+Asc is delivered in a lower volume and this likely contributed to the increased nausea reported by patients compared with the PEG+SPMC group. These results are consistent with other randomized studies of 1 L PEG+Asc,^12^, ^18^ and thereby confirm the validity of our study design and results.

Our study has several strengths. In addition to the randomized control study design, other factors that are well documented to affect bowel preparation quality such as split‐dosage and the pre‐colonoscopy diet were standardized across both groups. Previous studies have showed that morning colonoscopies demonstrate better bowel preparation quality when compared with afternoon colonoscopies.^32^, ^33^ This was taken into consideration in our study design and a separate subgroup analysis of morning and afternoon procedures was performed (Table 3). Thirdly, several individual blinded colonoscopists were involved, which negates the effect of individual bias on the subjective evaluation of bowel preparation quality.

An important limitation of our study is that it was terminated early due to the COVID‐19 pandemic and an unplanned interim analysis was performed. Early termination has resulted in an increase in the type I error (false positive), but has not affected the type II error for the primary endpoint. Second, there was room for potential selection bias during the recruitment process—patients were predominantly contacted for recruitment over the phone. Patients who were from a non‐English speaking background were less likely to be re‐contacted for recruitment with a phone interpreter. Furthermore, elderly and comorbid patients may have been less likely to personally attend telephone calls or consent to the study over the phone. This is evidenced by the relatively low median age of participants at 57 years. Furthermore, although participants and proceduralists were explicitly given instructions to not discuss the bowel preparation regimen, this could not be independently verified by the investigators and is a potential source of bias. We did not capture potential patient‐related risk factors for poor bowel preparation such as concurrent opioid or tricyclic antidepressant use, history of constipation, and comorbid conditions.^34^ However, in our center, patients who have previously been identified as having risk factors for poor bowel preparation are allocated an alternative extended bowel preparation regimen.

In conclusion, in this randomized study of 164 outpatients undergoing colonoscopy in an Australian tertiary center, the quality of bowel preparation achieved with 1 L PEG+Asc was non‐inferior to that achieved with PEG+SPMC. This is despite a lower volume of preparation (by 500 mL), which is required to be ingested with 1 L PEG+Asc. Furthermore, patient‐reported satisfaction and tolerability were similar for both 1 L PEG+Asc and PEG+SPMC, despite 1 L PEG+Asc causing more patient‐reported nausea. Further studies are required to see if these results can be replicated in subgroups such as elderly and comorbid patients and those patients with risk factors for suboptimal bowel preparation.
